# Manual of Physiology

**Published:** 1857-09

**Authors:** 


ART. IX.—*Manual of Physiology.* By WILLIAM SENHOUSE KIRKES, M.D., Fellow of the Royal College of Physicians, &c. A new and revised American, from the last London edition. With two hundred illustrations. Phildaelphia: Blanchard & Lea. 1857.

THIS excellent manual has been too long before the American medical public, and its merits are too well known, to require any special notice at our hands.

Confining himself strictly to physiology proper, and avoiding the many anatomical and chemical details, and the unsatisfactory discussions upon the as yet obscure and mooted points of the science, which encumber so many of the larger standard works, Dr. Kirkes has succeeded in giving to his book the character of a well-digested epitome, which the student will find a valuable guide during his attendance at college, and the busy practitioner a ready means to freshen his memory upon forgotten facts, or to keep pace with the science in its rapid advancement.

The present edition has undergone a careful revision. Various additions and alterations have been introduced into different parts, without, however, interfering, in any manner, with the general plan and arrangement of the book. The numerous and appropriate wood-cuts, with which it has been embellished by the American publishers, greatly enhance its value to the student.

